# Association Between Metabolic Syndrome and Asymptomatic Cerebral Arterial Stenosis: A Cross-Sectional Study in Shandong, China

**DOI:** 10.3389/fneur.2021.644963

**Published:** 2021-05-12

**Authors:** Shan Li, Xiao Sun, Yuanyuan Zhao, Xiang Wang, Xiaokang Ji, Shaowei Sang, Sai Shao, Yuanyuan Xiang, Guangbin Wang, Ming Lv, Fuzhong Xue, Qinjian Sun, Yifeng Du

**Affiliations:** ^1^Department of Neurology, Shandong Provincial Hospital, Cheeloo College of Medicine, Shandong University, Jinan, China; ^2^Department of Neurology, Shandong Provincial Hospital Affiliated to Shandong First Medical University, Jinan, China; ^3^Department of Biostatistics, School of Public Health, Shandong University, Jinan, China; ^4^Department of Clinical Epidemiology, Cheeloo College of Medicine, Qilu Hospital, Shandong University, Jinan, China; ^5^Department of Radiology, Shandong Provincial Hospital Affiliated to Shandong First Medical University, Jinan, China

**Keywords:** asymptomatic intracranial arterial stenosis, asymptomatic extracranial arterial stenosis, metabolic syndrome, asymptomatic cerebral arterial stenosis, stroke primary prevention

## Abstract

Metabolic syndrome (MetS) can worsen cerebral arterial atherosclerosis stenosis in patients with stroke; however, its effect on patients without stroke remains ambiguous. This study explored the association of MetS and its individual components with asymptomatic intracranial arterial stenosis (aICAS) and asymptomatic extracranial arterial stenosis (aECAS) among older Chinese adults. A total of 1988 participants from the Kongcun Town study aged ≥40 years and without a history of stroke were enrolled. The baseline data were obtained via face-to-face interviews. MetS was defined according to International Diabetes Federation criteria. Detection of aICAS was conducted using transcranial Doppler ultrasound, followed by diagnosis via magnetic resonance angiography. The evaluation of aECAS was performed using bilateral carotid ultrasonography. The aICAS and aECAS groups were 1:1 matched separately to the non-stenosis group by age and sex. The association between MetS and aICAS or aECAS was analyzed using multivariate logistic regression. Among the 1988 participants, 909 were diagnosed with MetS. The prevalence of MetS was higher in the aICAS group than in the non-stenosis group (*P* <0.001), but did not differ significantly between the aECAS and non-stenosis groups. The prevalence of aICAS increased with the number of MetS components from 3.4% in the ≤ 1 component group to 12.7% in the ≥4 components group (*P* for trend <0.001). After adjusting for confounding factors, MetS components associated with aICAS included central obesity, elevated triglyceride levels, and elevated blood pressure. None of the MetS components was associated with aECAS. MetS was positively associated with aICAS, but not with aECAS. Further, different components play different roles in the pathological process leading to aICAS.

## Introduction

Ischemic stroke caused by cerebral arterial atherosclerotic stenosis is a serious health and social issue worldwide, often leading to disability and mortality (1, 2). Some traditional vascular risk factors are involved in cerebral arterial atherogenesis, and they play different roles in the pathogenesis of intracranial and extracranial atherosclerosis (3). For instance, hyperlipidemia is more closely associated with extracranial arterial stenosis (ECAS), while diabetes mellitus (DM) and hypertension are associated with intracranial arterial stenosis (ICAS) (3, 4). Identifying different risk factors for ICAS and ECAS is helpful for efficient stroke prevention.

Metabolic syndrome (MetS) is a constellation of several metabolic risk factors, including central obesity, hypertension, elevated fasting blood glucose, and hyperlipidemia. The prevalence of MetS is gradually increasing with the aging population and changing lifestyle (5). In recent years, there has been a widespread concern regarding MetS as a novel risk factor for atherosclerosis (6).

MetS and its individual components are closely associated with ICAS or ECAS in patients with stroke, and this relationship is more significant with respect to ICAS than to ECAS (7–11). Some retrospective studies have evaluated the association of MetS with different locations (extracranial vs. intracranial) of asymptomatic cerebral arterial stenosis. A study in an asymptomatic Caucasians population found that MetS was an independent risk factor for moderate to severe (defined as ≥50% stenosis) intracranial atherosclerotic disease, but not for moderate to severe extracranial atherosclerotic disease (12). Another study involving a racially and ethnically diverse population found that the impact of MetS on the distribution of intracranial and extracranial atherosclerosis varied by race and ethnicity (13). Two community-based studies from China have separately reported a significant association between MetS and asymptomatic ICAS (aICAS) or asymptomatic ECAS (aECAS) (14, 15). However, no study has compared the association between MetS, including its individual components, and aICAS or aECAS in the Chinese population.

This study aimed to explore whether there is a differential profile in the association of MetS and its individual components with aICAS and aECAS among middle-aged and older adults living in rural communities in China.

## Materials and Methods

### Study Design and Population

This study was based on the Kongcun Town study (16), a population-based study targeting 2,311 rural residents aged ≥40 years with no history of clinical stroke. Data on demographics, medical history, and physical examinations were obtained through face-to-face interviews. aICAS was detected using a two-phase procedure: screening using transcranial Doppler and diagnosis via magnetic resonance angiography. aECAS was evaluated using bilateral carotid ultrasonography. Among the 2,311 participants, 305 were excluded owning to incomplete information, two because of abnormal waist circumference, and 16 with combined aICAS/aECAS were excluded owning to the small number of cases. Finally, data from the 1988 eligible participants were analyzed. Participants were categorized into the following three groups according to the site of stenosis: (1) non-stenosis (*n* = 1813) (2) isolated aICAS (*n* = 132); and (3) isolated aECAS (*n* = 43).

The study protocol was approved by the Ethical Standards Committee on Human Experimentation at Shandong Provincial Hospital, Cheeloo College of Medicine, Shandong University. This study was conducted in accordance with the principles of the Declaration of Helsinki. All participants provided a written informed consent.

### Definitions of Vascular Risk Factors

Baseline data on demographics and risk factors were collected via interviews, clinical examinations, and laboratory tests in a similar manner as reported in our previous study (16). Hypertension was defined as a systolic blood pressure of ≥140 mm Hg, diastolic blood pressure of ≥90 mm Hg, use of antihypertensive drugs, or self-reported hypertension. DM was defined as a fasting plasma glucose level of ≥7.0 mmol/L (126.0 mg/dL), use of blood glucose-lowering drugs, receipt of insulin injection, or a self-reported history of diabetes. Based on smoking habits, participants were classified into current smokers (smoked at least one cigarette per day for more than 1 year) and former smokers (quit smoking <6 months earlier). Based on their drinking habits, participants were classified into current drinkers (consumed alcohol at least once a week for at least 6 months) and former drinkers (quit <6 months earlier).

### Definition of MetS

MetS was defined using the criteria previously published by the International Diabetes Federation (17). The definition included the presence of central obesity (waist circumference ≥90 cm for Chinese men and ≥80 cm for Chinese women), plus any two of the following: (1) triglyceride (TG) level ≥1.7 mmol/L (150 mg/dL) or receiving specific treatment for this lipid abnormality (2) high-density lipoprotein cholesterol (HDL-C) level <1.03 mmol/L (40 mg/dL) in men and <1.29 mmol/L (50 mg/dL) in women or receiving specific treatment for this lipid abnormality; (3) systolic blood pressure ≥130 mm Hg or diastolic blood pressure ≥85 mm Hg or receiving treatment for previously diagnosed arterial hypertension; and (4) fasting plasma glucose level ≥5.6 mmol/L (100 mg/dL) or previously diagnosed DM.

### Assessment of aICAS and aECAS

The protocol for the evaluation of aICAS and aECAS has been described in detail in our previous study (16). In brief, aICAS was detected through a two-phase procedure: a screen phase using transcranial Doppler and the diagnostic phase using magnetic resonance angiography. Transcranial Doppler was performed by two physicians using a portable machine (VIASYS Companion III). The bilateral middle cerebral artery, internal carotid artery, anterior cerebral artery, posterior cerebral artery, vertebral artery, and basilar artery were examined with a 2-MHz probe via temporal, occipital, and eye windows. Stenosis of participants with poor temporal windows in the bilateral vertebral artery, posterior cerebral artery, and basilar artery was examined via the occipital window. In this study, aICAS was defined according to the previously published criteria for the identification of one or more stenotic lesions of any degree in any one of the analyzed intracranial vessels on magnetic resonance angiography (18). The diagnosis and grading of aICAS was performed by a stroke specialist and clinical neuroradiologist. The degree of stenosis in the evaluated arteries (bilateral middle cerebral artery, bilateral intracranial internal carotid artery, anterior cerebral artery, posterior cerebral artery, and basilar artery) was classified into five grades by consensus as normal, mild (signal reduction <50%), moderate (signal reduction ≥50% and <70%), severe (signal reduction ≥70%), or occlusion (focal signal loss with the presence of distal signal). aECAS was diagnosed via carotid ultrasonography examination, which has a high sensitivity and specificity (19, 20), and was performed by two experienced physicians. aECAS was defined according to established carotid ultrasonography criteria (21) as the identification of one or more stenotic lesions of any degree in any one of the analyzed vessels, including the carotid artery, internal carotid artery, and external carotid artery. The degree of aECAS was classified into four grades: mild (<50% stenosis), moderate (50–69% stenosis), severe (70–99% stenosis), and total occlusion.

### Statistical Analyses

All analyses were conducted using IBM Statistical Package for the Social Sciences Statistics V22.0 for Windows (IBM Corp., released 2013, Armonk, NY, USA). Baseline population statistics and continuous laboratory-based variables are expressed as terms of mean and standard deviation, and categorical variables are expressed as frequencies and percentages. Continuous variables were compared using the *t*-test or analysis of variance with *post hoc* tests, while categorical variables were compared using the chi-square test. The Bonferroni adjustment was performed to assess the statistical significance of the intergroup differences. The aICAS and aECAS groups were 1:1 matched separately to the non-stenosis group by age and sex. The multivariate logistic regression was used to determine the association between MetS and its individual components with aICAS or aECAS. The variables with a *P*-value of <0.1 in the univariate analysis were included in the logistic regression models. The associations of different cerebral arterial stenosis were reported as odds ratio (OR) values and their 95% confidence intervals (CI). All statistical tests were two-tailed, and *P* < 0.05, indicated statistical significance.

## Results

### Baseline Characteristics of the Study Population

The demographic and clinical characteristics of the study participants are shown in Table 1. Compared with the non-stenosis group, the aICAS group had significantly higher mean age, body mass index, waist circumference, and TG and low-density lipoprotein cholesterol levels and a significantly lower mean HDL-C level. The prevalence of hypertension, DM, and MetS was also higher in the aICAS group than in the non-stenosis group. The mean age and prevalence of hypertension and DM were also higher in the aECAS group than in the non-stenosis group. Of the 1988 participants, 909 (45.7%) were diagnosed with MetS. Compared with the non-stenosis group, the prevalence of MetS and all its individual components (all *P* < 0.017) was higher in the aICAS group and that of elevated blood pressure and elevated fasting glucose (all *P* < 0.017) was higher in the aECAS group.

**Table 1:** Demographic and clinical characteristics of study participants.

**Characteristics**	**Overall (*n* = 1,988)**	**NS (*n* = 1,813)**	**aICAS (*n* = 132)**	**aECAS (*n* = 43)**	**OverallP**
Age (years), mean (SD)	57.6 (10.3)	57.2 (10.3)	60.3 (10.8)^a^	66.6 (8.1)^a^	<0.001
Male, *n* (%)	956 (48.0)	880 (48.5)	51 (38.6)	25 (58.1)	0.037
Hypertension, *n* (%)	1148 (57.7)	1004 (55.3)	110 (83.3)^a^	34 (79.0)^a^	<0.001
Diabetes mellitus, *n* (%)	304 (15.2)	250 (13.7)	30 (35.7)^a^	15 (34.8)^a^	<0.001
Total cholesterol (mmol/l), mean (SD)	5.4 (1.0)	5.4 (1.0)	5.4 (1.0)	5.6 (1.0)	0.265
Triglycerides (mmol/l), mean (SD)	1.4 (0.9)	1.3 (0.9)	1.7 (1.2)^a^	1.5 (1.1)	<0.001
HDL-C (mmol/l), mean (SD)	1.6 (0.4)	1.6 (0.4)	1.5 (0.3)^a^	1.7 (0.4)	<0.001
LDL-C (mmol/l), mean (SD)	3.0 (0.7)	3.0 (0.7)	3.2 (0.7)^a^	3.1 (0.7)	0.013
Smoking habits, *n* (%)	448 (22.5)	420 (23.1)	15(11.3)^a^	13 (30.2)	0.004
Drinking habits, *n* (%)	658 (33.0)	608 (33.5)	35 (26.5)	15 (34.8)	0.246
BMI (kg/m^2^), mean (SD)	25.1 (3.3)	25.1 (3.4)	26.2 (3.0)^a^	24.6 (3.3)	0.001
Waist circumference (cm), mean (SD)	91 (9)	91 (9)	95 (8)^a^	91 (10)	<0.001
MetS, *n* (%)	909 (45.7)	788 (43.4)	95 (71.9)^a^	26 (60.4)	<0.001
**MetS components**					
Central obesity, *n* (%)	1547 (77.8)	1389 (76.6)	126 (95.4)^a^	32 (74.4)	<0.001
Raised triglycerides, *n* (%)	448 (22.5)	392(21.6)	45 (34.0)^a^	11 (25.5)	0.004
Reduced HDL-C, n (%)	217 (10.9)	188 (10.3)	25 (18.9)^a^	4 (9.3)	0.009
Raised BP, *n* (%)	1578 (79.3)	1419 (78.2)	118 (89.3)^a^	41 (95.3)^a^	<0.001
Elevated fasting glucose, *n* (%)	1062 (53.4)	944 (52.0)	86(65.1)^a^	32 (74.4)^a^	<0.001
Number of MetS component	2 (1)	2 (1)	3 (1)^a^	3 (1)	<0.001

*NS, non-stenosis; aICAS, asymptomatic intracranial arterial stenosis; aECAS, asymptomatic extracranial arterial stenosis; LDL-C, low-density lipoprotein cholesterol; HDL-C, high-density lipoprotein cholesterol; BMI, body mass index; BP, blood pressure; MetS, metabolic syndrome; SD: standard deviation*.

a *Significantly different from NS group, P < 0.05/3 = 0.017 (the Bonferroni correction was applied)*.

*Overall p-value is for the test of difference among the NS group, aICAS group and aECAS group*.

### Demographic and Clinical Characteristics of the Participants After Matching for Age and Sex

Table 2 shows the demographic and clinical characteristics of the participants after matching for age and sex. Age, sex, smoking habits, and drinking habits showed no significant difference between the aICAS/aECAS and control groups. Compared with the control 1 group, the aICAS group had significantly higher body mass index, waist circumference, and a significantly lower mean HDL-C level. The prevalence of hypertension and MetS was also higher in the aICAS group than in the control 1 group. The HDL-C level was lower in the aECAS group than in the control 2 group.

**Table 2:** Demographic and clinical characteristics of the participants after matching for age and sex.

**Characteristics**	**Control 1 (*n* = 132)**	**aICAS (*n* = 132)**	**P^**a**^**	**Control 2 (*n* = 43)**	**aECAS (*n* = 43)**	**P^**b**^**
Age (years), mean (SD)	58.4 (10.6)	60.3 (10.8)	0.157	64.7 (8.0)	66.6 (8.1)	0.276
Male, *n* (%)	51 (38.6)	51 (38.6)	1.000	25 (58.1)	25 (58.1)	1.000
Hypertension, *n* (%)	84 (63.6)	110 (83.3)	<0.001	30 (69.8)	34 (79.0)	0.323
Diabetes mellitus, *n* (%)	32 (24.2)	39 (29.5)	0.331	15 (34.9)	15 (34.9)	1.000
Total cholesterol (mmol/l), mean (SD)	6.9 (1.0)	5.4 (1.0)	<0.001	7.1 (0.9)	5.6 (1.0)	<0.001
Triglycerides (mmol/l), mean (SD)	1.8 (1.2)	1.7 (1.2)	0.419	1.8 (1.3)	1.5 (1.1)	0.312
HDL-C (mmol/l), mean (SD)	1.7 (0.4)	1.5 (0.3)	<0.001	1.9 (0.4)	1.7 (0.4)	0.003
LDL-C (mmol/l), mean (SD)	3.9 (0.7)	3.2 (0.7)	<0.001	3.9 (0.8)	3.1 (0.7)	<0.001
Smoking habits, *n* (%)	22 (16.7)	15(11.3)	0.215	17(39.5)	13 (30.2)	0.365
Drinking habits, *n* (%)	34 (25.8)	35 (26.5)	0.889	18 (41.9)	15 (34.8)	0.506
BMI (kg/m^2^), mean (SD)	24.9 (3.5)	26.2 (3.0)	0.002	23.2 (3.7)	24.6 (3.3)	0.059
Waist circumference (cm), mean (SD)	92 (10)	95 (8)	0.021	87 (10)	91 (10)	0.058
MetS, *n* (%)	78 (53.8)	95 (71.9)	0.002	19 (44.2)	26 (60.4)	0.131
Central obesity, *n* (%)	108 (81.8)	126 (95.4)	<0.001	24 (55.8)	32 (74.4)	0.070
Raised triglycerides, *n* (%)	55(41.7)	45 (34.0)	0.205	17(39.5)	11 (25.5)	0.167
Reduced HDL-C, *n* (%)	11 (8.3)	25 (18.9)	0.012	3(7.0)	4 (9.3)	0.693
Raised BP, *n* (%)	109 (82.6)	118 (89.3)	0.111	35(81.4)	41 (95.3)	0.044
Elevated fasting glucose, *n* (%)	89(67.4)	86(65.1)	0.696	32 (74.4)	32 (74.4)	1.000
Number of MetS component	3 (1)	3 (1)	0.105	3 (1)	3 (1)	0.389

*aICAS, asymptomatic intracranial arterial stenosis; aECAS, asymptomatic extracranial arterial stenosis; LDL-C, low-density lipoprotein cholesterol; HDL-C, high-density lipoprotein cholesterol; BMI, body mass index; BP, blood pressure; MetS, metabolic syndrome*.

*P^a^ value is for the test of difference between the control 1 group and aICAS group*.

*P^b^ value is for the test of difference between the control 2 group and aECAS group*.

### Associations of MetS and Its Components With aICAS or aECAS

**Figure 1:**
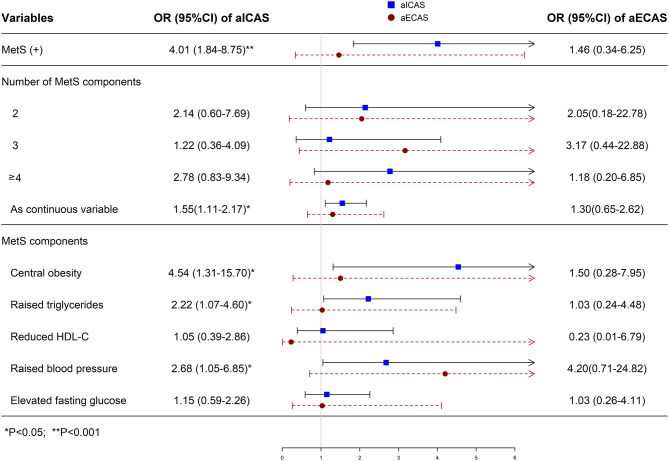
Multivariate logistic regression analysis of the association of MetS with aICAS or aECAS. aICAS, asymptomatic intracranial arterial stenosis; aECAS, asymptomatic extracranial arterial stenosis; LDL-C, low-density lipoprotein cholesterol; HDL-C, high-density lipoprotein cholesterol; BMI, body mass index; BP, blood pressure; MetS, metabolic syndrome; OR, odds ratio. ORs were calculated using logistic regression model after adjusting total cholesterol, BMI, and LDL-C.

In the multivariate logistic regression analysis (Figure 1), MetS was significantly associated with aICAS (OR: 4.01; 95% CI: 1.84, 8.75) after adjusting for total cholesterol level, body mass index, and low-density lipoprotein cholesterol level, which were significantly related to aICAS or ECAS in the univariate logistic regression analysis (Table 3, all *P* <0.1). Participants with more severe MetS components were more likely to have aICAS (*P* for linear trend =0.011). The following MetS components were significantly associated with aICAS: central obesity, elevated TG levels, and elevated blood pressure. However, no significant association between aECAS, MetS and its components was observed.

**Table 3:** Univariate logistic regression analysis of the association of MetS with aICAS or aECAS.

**Variables**	**aICAS**		**aECAS**	
	**Odds ratio (95% CI)**	***p***	**Odds ratio (95% CI)**	***p***
Smoking habits	1.56 (0.77–3.16)	0.217	1.51 (0.62–3.69)	0.367
Drinking habits	1.04 (0.60–1.80)	0.889	1.34 (0.56–3.21)	0.506
BMI	1.13 (1.04–1.22)	0.003	1.13 (0.99–1.29)	0.064
Total cholesterol	3.89 (2.82–5.58)	<0.001	6.90 (3.02–15.87)	<0.001
LDL-C	4.13 (2.75–6.25)	<0.001	5.85 (2.53–13.51)	<0.001
MetS (+)	2.22 (1.32–3.68)	0.002	1.93 (0.81–4.56)	0.132
**Number of MetS components**				
≤ 1	Reference		Reference	
2	1.11 (0.44–2.80)	0.830	1.44 (0.34–6.05)	0.618
3	1.21 (0.49–3.00)	0.677	3.15 (0.86–11.60)	0.084
≥4	2.62 (1.01–6.80)	0.049	1.35 (0.34–5.44)	0.673
As continuous variables	1.21 (0.96–1.52)	0.105	1.19 (0.81–1.74)	0.385
**MetS components**				
Central obesity	4.67 (1.84–11.84)	0.001	2.30 (0.93–5.73)	0.073
Raised triglycerides	0.72 (0.44–1.19)	0.205	1.90 (0.76–4.76)	0.170
Reduced HDL-C	2.57 (1.21–5.47)	0.014	1.37 (0.29–6.51)	0.694
Raised BP	1.78 (0.87–3.63)	0.114	4.69 (0.93–23.53)	0.061
Elevated fasting glucose	0.90 (0.54–1.51)	0.696	1.00 (0.38–2.64)	1.000

*aICAS, asymptomatic intracranial arterial stenosis; aECAS, asymptomatic extracranial arterial stenosis; LDL-C, low-density lipoprotein cholesterol; HDL-C, high-density lipoprotein cholesterol; BMI, body mass index; BP, blood pressure; MetS, metabolic syndrome*.

### Prevalence of aICAS and aECAS According to the Number of MetS Components

**Figure 2:**
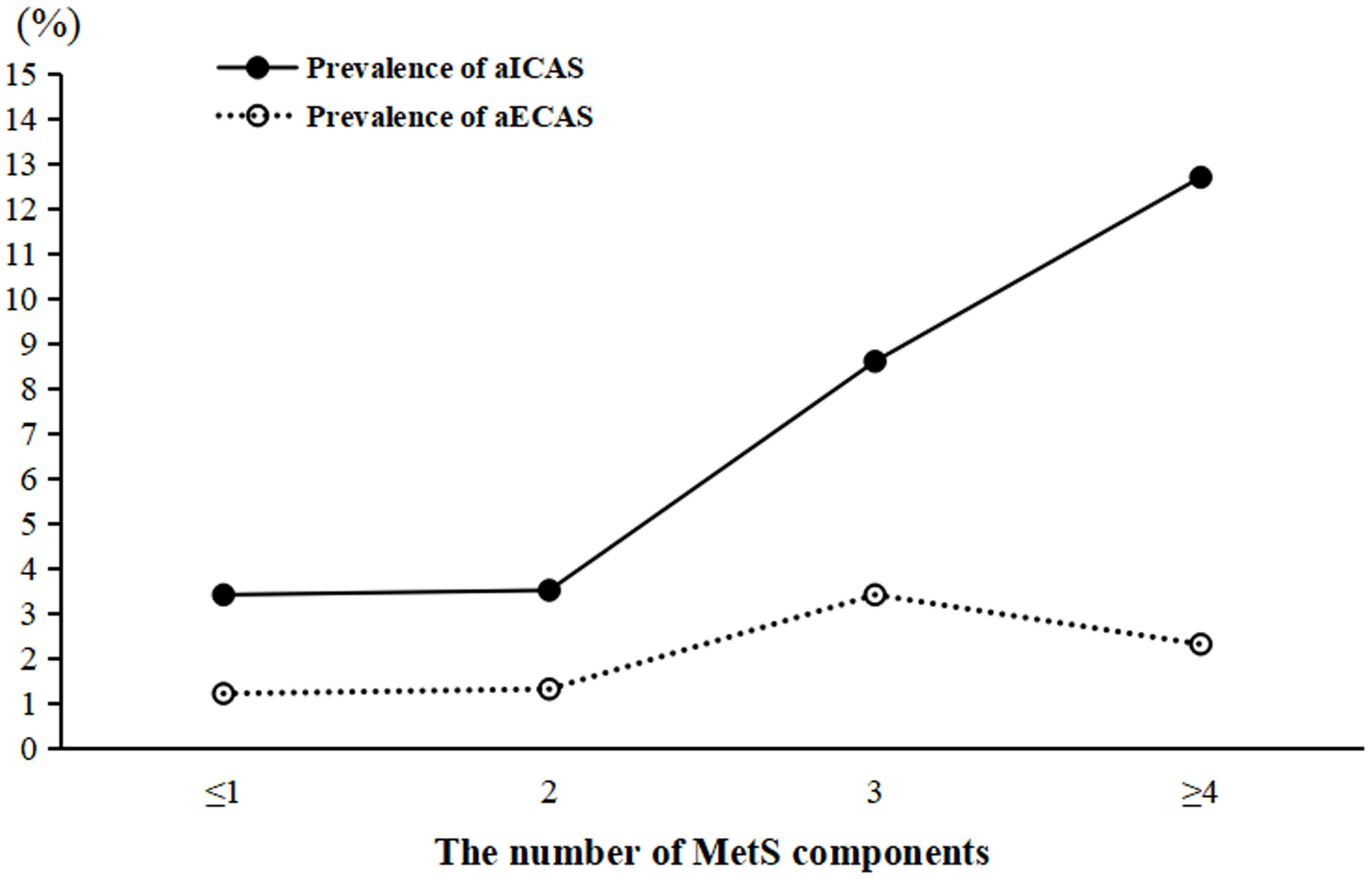
Prevalence of aICAS and aECAS according to the number of MetS components. aICAS, asymptomatic intracranial arterial stenosis; aECAS, asymptomatic extracranial arterial stenosis; MetS, metabolic syndrome.

The prevalence of aICAS increased significantly in proportion to the number of MetS components from 3.4% in the ≤ 1 component group to 12.7% in the ≥4 components group (*P* for trend <0.001). However, the same association was not found for aECAS (Figure 2).

## Discussion

This study found that MetS was associated with aICAS, but not with aECAS, and different components play different roles in the pathological process leading to aICAS. Among MetS components, central obesity, elevated TG levels, and elevated blood pressure were significantly associated with aICAS. To the best of our knowledge, this is the first study to investigate the association between MetS and aICAS or aECAS among middle-aged and older adults living in rural communities in China.

MetS is a proinflammatory and hypercoagulable state, which is mainly mediated by insulin resistance. It has been suggested that accelerated atherosclerosis in MetS is associated with defective insulin signaling pathways (22). In addition, oxidative stress is associated with MetS and plays a role in endothelial dysfunction and subsequent atherosclerosis (23). Previous hospital-based studies reported that MetS was an independent risk factor for stroke, and that patients with MetS were more likely to experience ICAS than ECAS (7–11). A prospective Korean study revealed that MetS was more prevalent in ICAS patients than in ECAS patients with posterior circulation stroke (3). These studies concluded that MetS could play an important role in promoting cerebral arterial stenosis and increasing the risk of subsequent stroke. Furthermore, several studies have focused on investigating the association between MetS and aICAS or aECAS in the stroke-free population. The Asymptomatic Polyvascular Abnormalities in Community study targeting employees and retirees of the Kailuan (Group) Co. Ltd (a large coal mine industry) reported significant associations between MetS and aICAS (14), which is consistent with our results. Another study involving asymptomatic Chinese people reported significant associations between MetS and aECAS in menopausal women (15). The reason for the inconsistent results may be that the participants investigated were different. Regarding severity, the Barcelona-Asymptomatic Intracranial Atherosclerosis study found that MetS was significantly associated with moderate to severe intracranial atherosclerotic disease and not with moderate to severe extracranial atherosclerotic disease (12). This finding was apparently corroborated by the finding that MetS may be independently associated with the early stage rather than the advanced stages of intracranial arterial atherosclerosis (24). To the best of our knowledge, some studies have reported that ECAS is common in Caucasian population, whereas ICAS is more frequent in Asian and African populations (25–27). A previous study involving a racially and ethnically diverse population found that the impact of MetS on the distribution of intracranial and extracranial atherosclerosis varied by race and ethnicity (13). The race-specific impact of MetS on the distribution of cerebral arterial atherosclerosis may be caused by racial-specific differences in the prevalence of MetS components and in the host response to the presence of specific components. This finding may partly explain the well-known differences in race-ethnic predilection to intracranial or extracranial atherosclerosis. Future studies with genotyping may be important to define the relationships between the biological race differences between ICAS and ECAS.

In this study, central obesity was associated with aICAS, but not with aECAS. Central obesity can lead to an increase in the free fatty acids, which play an important role in the pathogenesis of insulin resistance (28, 29). Furthermore, insulin resistance can damage intracranial vasodilatory function by increasing the stiffness of the vascular wall and reducing the buffering ability, which increases its susceptibility to arterial oxidative stress (30, 31). Therefore, early intervention for central obesity may delay the progression of cerebral arterial stenosis, especially aICAS.

In this study, elevated TG levels were significantly associated with aICAS. High TG levels can promote the formation of low-density lipoprotein particles (32); high levels of low-density lipoprotein cholesterol, especially its oxidized form, can facilitate endothelial dysfunction, which is the first step in atherosclerotic plaque formation (33).

The significant association between elevated blood pressure and aICAS detected in this study is consistent with previous studies (34, 35). Hypertension is a well-known risk factor for arteriosclerosis (36). Compared with the extracranial arteries, the thickness and elasticity of the tunica media is inferior in the intracranial arteries; therefore, it may be more vulnerable to the changes in vascular stress and blood flow caused by hypertension (36).

Among MetS components, the association between reduced HDL-C and elevated fasting glucose levels with MetS was not found. However, previous hospital-based studies found that the components (reduced HDL-C and elevated fasting glucose levels) constituting MetS were related to aICAS (9, 11). This suggests that MetS can affect intracranial arterial atherosclerosis in different pathological states via different metabolic pathways. In addition, this finding may reveal that the association between aICAS and MetS may be derived from the specific components of MetS, such as central obesity, elevated TG levels, and elevated blood pressure, especially in the asymptomatic phase of intracranial arterial stenosis. More metabolism-related basic research is needed to confirm this inference in the future.

The reasons for the aforementioned differential effects of MetS on the distribution of cerebral arterial stenosis are not well-understood. The potential reasons for this are as follows: first, the differential responses of intracranial and extracranial arteries to oxidative stress may explain our finding that most components constituting MetS were associated with aICAS, since oxidative stress has been reported to be associated with MetS (23). Compared with the extracranial arteries, the intracranial arteries were found to be susceptible to oxidative stress with increasing age (37). Second, the histological differences between the intracranial and extracranial arteries should be considered. The extracranial arteries are elastic arteries whose tunica media are rich in elastin filaments. However, the intracranial arteries with a fewer elastic fibers may be more vulnerable to the circulatory abnormalities caused by MetS (36).

Some potential limitations of our study are worth mentioning. First, a cross-sectional study cannot prove the existence of a causal relationship between MetS and aICAS/aECAS; further studies using a prospective study are needed to confirm this relationship. Second, owning to the relatively small sample size, this study was unable to evaluate the association between MetS and distribution of stenosis in various strata of severity of stenosis. Finally, the findings of this study may not be generalizable to other populations since it included only Chinese adults living in rural areas. Nevertheless, to the best of our knowledge, this is the first study to investigate the differences in the associations between certain MetS components and the distribution of cerebral arterial stenosis.

In conclusion, the study findings indicate that MetS is associated with aICAS, but not with aECAS, and different components play different roles in the pathological process of aICAS. These differences may prompt the employment of individualized preventive measures during the asymptomatic stage of cerebral arterial stenosis; thereby, reducing the incidence of stroke.

## Data Availability Statement

The raw data supporting the conclusions of this article will be made available by the authors, without undue reservation.

## Ethics Statement

The studies involving human participants were reviewed and approved by Shandong Provincial Hospital, Cheeloo College of Medicine, Shandong University. The patients/participants provided their written informed consent to participate in this study.

## Author Contributions

QS, YD, and FX conceived and designed the research. SL, YZ, XW, XJ, SSa, SSh, and YX acquired the data. SL, XS, YZ, XW, XJ, SSa, SSh, YX, and GW analyzed and interpreted the data. SL and XS draft the manuscript. XW, ML, FX, QS, and YD made critical revisions of the manuscript. All authors approved the final manuscript.

## Conflict of Interest

The authors declare that the research was conducted in the absence of any commercial or financial relationships that could be construed as a potential conflict of interest.
